# Cell-penetrating peptide-conjugated copper complexes for redox-mediated anticancer therapy

**DOI:** 10.3389/fphar.2022.1060827

**Published:** 2022-11-15

**Authors:** Quim Peña, Sergi Rodríguez-Calado, A. Jalila Simaan, Mercè Capdevila, Pau Bayón, Oscar Palacios, Julia Lorenzo, Olga Iranzo

**Affiliations:** ^1^ Departament de Química, Facultat de Ciències, Universitat Autònoma de Barcelona, Barcelona, Spain; ^2^ Aix Marseille University, CNRS, Centrale Marseille, ISm2, Marseille, France; ^3^ Department of Nanomedicine and Theranostics, Institute for Experimental Molecular Imaging, Faculty of Medicine, RWTH Aachen University Clinic, Aachen, Germany; ^4^ Department Bioquímica i Biologia Molecular, Institut de Biotecnologia i Biomedicina, Universitat Autònoma de Barcelona, Barcelona, Spain

**Keywords:** cell-penetrating peptide, copper, metal complex, intracellular delivery, cancer, redox-active, metallodrug

## Abstract

Metal-based chemotherapeutics like cisplatin are widely employed in cancer treatment. In the last years, the design of redox-active (transition) metal complexes, such as of copper (Cu), has attracted high interest as alternatives to overcome platinum-induced side-effects. However, several challenges are still faced, including optimal aqueous solubility and efficient intracellular delivery, and strategies like the use of cell-penetrating peptides have been encouraging. In this context, we previously designed a Cu(II) scaffold that exhibited significant reactive oxygen species (ROS)-mediated cytotoxicity. Herein, we build upon the promising Cu(II) redox-active metallic core and aim to potentiate its anticancer activity by rationally tailoring it with solubility- and uptake-enhancing functionalizations that do not alter the ROS-generating Cu(II) center. To this end, sulfonate, arginine and arginine-rich cell-penetrating peptide (CPP) derivatives have been prepared and characterized, and all the resulting complexes preserved the parent Cu(II) coordination core, thereby maintaining its reported redox capabilities. Comparative *in vitro* assays in several cancer cell lines reveal that while specific solubility-targeting derivatizations (i.e., sulfonate or arginine) did not translate into an improved cytotoxicity, increased intracellular copper delivery *via* CPP-conjugation promoted an enhanced anticancer activity, already detectable at short treatment times. Additionally, immunofluorescence assays show that the Cu(II) peptide-conjugate distributed throughout the cytosol without lysosomal colocalization, suggesting potential avoidance of endosomal entrapment. Overall, the systematic exploration of the tailored modifications enables us to provide further understanding on structure-activity relationships of redox-active metal-based (Cu(II)) cytotoxic complexes, which contributes to rationalize and improve the design of more efficient redox-mediated metal-based anticancer therapy.

## Graphical Abstract

**Figure:**
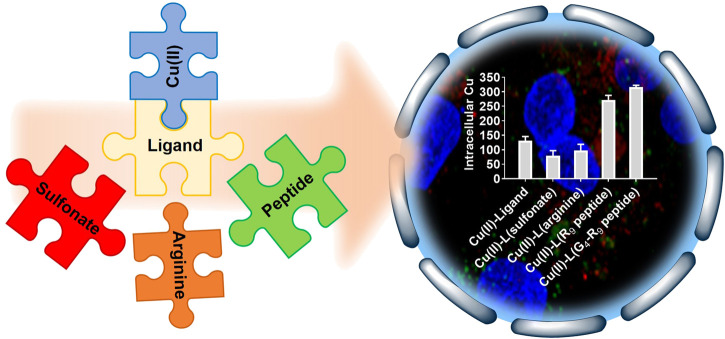
Modulation of solubility and intracellular delivery of redox-active copper complexes via cell-penetrating peptide-conjugation enhances cytotoxicity.

## Introduction

Metal-based chemotherapeutics have shown high clinical relevance in cancer therapy due to the possibility of tackling diverse (sub)cellular targets and acting *via* multiple mechanisms of action thanks to the unique metal-ligand interplay (Anthony et al., 2020). Platinum (Pt) complexes such as cisplatin, carboplatin and oxaliplatin have already become first-line treatments for several types of cancer (Rottenberg et al., 2021). Despite their success, the high reactivity, lack of specificity and non-physiological nature of Pt often lead to undesired biological interactions and side-effects (de Luca et al., 2019; Ohmichi et al., 2005; Oun et al., 2018; Wheate et al., 2010; Zhou et al., 2020). To overcome some of these obstacles, the use of metal complexes bearing physiological metals such as copper (Cu) have arisen as promising alternatives. In addition to the enhanced biocompatibility of Cu, its complexes can induce toxicity *via* diverse mechanisms beyond the common DNA-binding mode of action of Pt(II) complexes, such as DNA cleavage, topoisomerase inhibition and oxidative (mitochondrial) disruption (Santini et al., 2014). In particular, the different biologically attainable oxidation states of Cu enable the preparation of what is broadly known as catalytic metallodrugs, i.e., metal complexes that mediate catalytic reactions in biological environments, including the generation of reactive oxygen species (ROS).

ROS-based chemotherapy has awakened extensive interest due to the possibility of promoting selectivity towards cancer cells, which is based on the higher basal ROS levels in cancer than in healthy cells (Liou and Storz, 2010), and of bypassing potential drug resistance mechanisms (Cui et al., 2018). Additionally the generally more reducing environment of cancer cells (Petrova et al., 2018) can favor the reduction of Cu(II), thereby triggering the catalytic cycle. Several reported Cu (and other metal) complexes have already shown promising therapeutic performance through ROS-based mechanisms of action (Li et al., 2020; Liu et al., 2019; Peña et al., 2019; Simunkova et al., 2019; Sîrbu et al., 2017). Nonetheless, most Cu- and other metal-related biological pathways, including catalytic processes to generate radical species, occur, to a considerable extent, at the intracellular level. Additionally, metal complexes do often face solubility issues that contribute to limit its real applicability (Savjani et al., 2012). Hence, and given that many chemotherapeutics are administered intravenously, solubility and intracellular delivery appear as two important features in the design of potential redox-active (Cu) chemotherapeutics.

To help overcome these challenges, the use of delivery systems or functional moieties that increase solubility and enhance intracellular delivery, such as viral-based vectors, vesicles, nanoparticles, or cell-penetrating peptides (CPPs) can be used (Allen and Cullis, 2004; Dhar et al., 2009; Nakase et al., 2012; Stewart et al., 2018; Tran et al., 2017; Vargason et al., 2021; Zhou & Kopeček, 2013) Concretely, the latter is one of the most common and promising strategies to deliver therapeutics inside cancer cells. CPPs comprise a class of short peptides (<30 amino acids) that have the ability to cross cellular membranes (Schmidt et al., 2010; Guidotti et al., 2017). They have acquired considerable attention due to their high transduction efficiency and low associated cytotoxicity (Guo et al., 2016; Guidotti et al., 2017; Borrelli et al., 2018). More than 80% of the known CPPs are cationic and contain more than five positively charged amino acids, being arginine (Arg)-rich CPPs the most widely studied class by far (Schmidt et al., 2010; Milletti, 2012; Raucher and Ryu, 2015). Multiple mechanisms, highly dependent on the physicochemical nature and number of amino acid residues, have been reported for CPPs, including translocation, diffusion and endocytosis (Brock, 2014). The positively charged guanidinium groups in the arginine residues have high affinity for the externally-faced negatively charged fatty acids of the cell membrane, mediating the peptide insertion into the cytosol (Rothbard et al., 2004; Herce et al., 2014). One of the key issues in efficient intracellular delivery of CPPs is the subsequent endosomal escape. Most of the reported CPPs are able to promote internalization, but the mechanisms for which they can escape from endosomes are still not completely understood. Among them, strategies encompassing the use of cationic structures (such as Arg-rich based) and/or amphiphilic structures that can disrupt endosomal membranes because of the high electrostatic charge and partial hydrophobicity have been reported (Brock et al., 2018; Najjar et al., 2017).

Taking all into account, we here aim to (1) explore feasible, rational and biologically relevant functionalization strategies that tackle the solubility and intracellular delivery issues that many (redox-active) Cu complexes face while, at the same time, (2) systematically assessing (and eventually rationalizing) the impact of these specific solubility- and uptake-promoting modifications in the activity of the complex against cancer cells. To evaluate this, we built upon a Cu(II) *N,N,O*-chelating salphen-like complex (C1), recently reported by our group (Peña et al., 2021). Despite exhibiting some solubility issues, C1 was observed to display a remarkable redox-mediated cytotoxicity, and a putative discrimination between cancer and healthy cells, through the generation of ROS. To this end, we established simple, modular and versatile functionalization methodologies to prepare several tailored derivatives of the parent ligand of the complex **C1** (i.e., **H**_**2**_ **L1**), with ideally no or minimal alteration of the metal-ligand interaction, known to play a key role in determining the final (re)activity.

**FIGURE 1:**
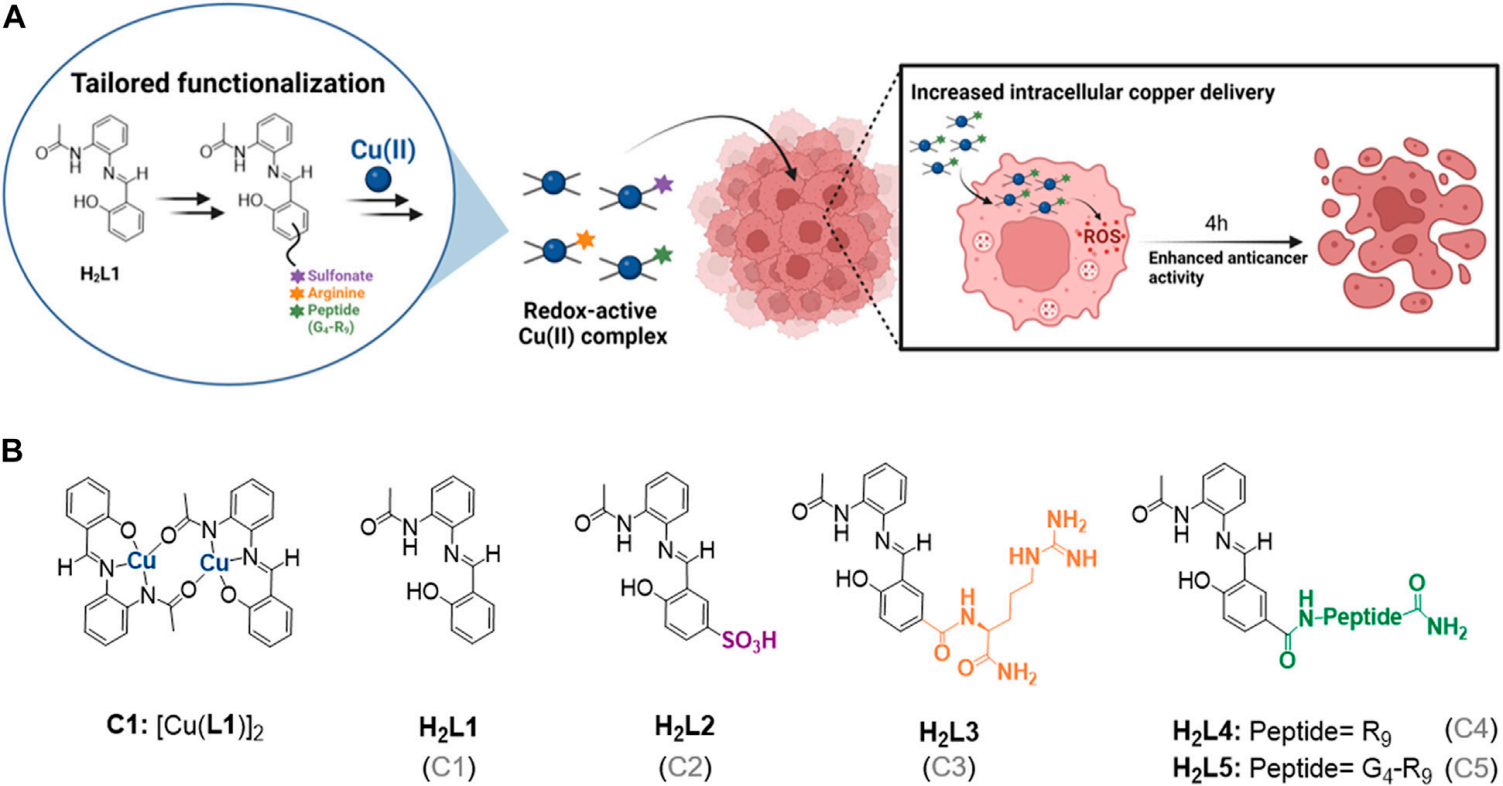
**(A)** Schematic representation of the work rationale. Assessing the impact of the different functionalizations on the uptake and anticancer activity of Cu(II) complexes. **(B)** Structure of the Cu(II) complex C1 (Peña et al., 2021) and the different H_2_L1 functionalized ligands (H_2_L2-H_2_L5), where R and G in the peptides structure correspond to arginine (Arg) and glycine amino acids, respectively. The name of the Cu(II) complex corresponding to each ligand is specified in parentheses.

The rationale of our work is schematically depicted in Figure 1A and the specific derivatizations shown in Figure 1B. First, two different functionalization strategies were explored with the main goal of increasing solubility in aqueous media, namely the addition of a sulfonate group (ligand H_2_L2 and complex C2), and of an arginine (Arg) residue (ligand H_2_L3 and complex C3) (Figure 1B). Both groups are biologically relevant and introduce a charge of opposite sign at physiological pH, i.e., negative for sulfonate group and positive for Arg. Taking into account that the number of Arg residues affects the internalization ability of CPPs (being those with at least six Arg residues more efficient (Wender et al., 2000; Tünnemann et al., 2008; Raucher and Ryu, 2015; Guidotti et al., 2017)), and in order to tackle potential cellular uptake limitations as well, two additional modifications were also evaluated: a nine Arg residues peptide (R_9_, ligand H_2_L4 and complex C4) and its analogous sequence with four glycine (G) residues (G_4_-R_9_, ligand H_2_L5 and complex C5) (Figure 1B). R_9_ was selected for its well-known good cell-penetrating capabilities (Wender et al., 2000; Guidotti et al., 2017), and glycine residues were introduced to test the effect of having a linker between the CPP and the metallic core. Additionally, the cationic nature of the CPPs, in combination with the relatively more hydrophobic salphen-based metallic core, may help address possible endo-/lysosomal entrapment issues (Brock et al., 2018). Whilst using here C1 as our model parent scaffold, we expect that the obtained data will contribute to further rationalize the role of the different functionalities in tuning the intracellular delivery and corresponding activity of analog redox-active Cu(II) complexes, overall providing a starting point for the design of future improved ROS-inducing (Cu) metallodrugs.

## Materials and methods

### Chemicals and reagents

Reagents like copper(II) chloride, copper(II) acetate, sodium carbonate, sodium sulfate, hydrochloric acid, ammonium chloride, *o-*phenylenediamine, 2-amino-2-(hydroxymethyl)propane-1,3-diol (TRIS), *N,N*-diisopropylethylamine (DIEA), triisopropylsilane (TIS), *N-*methyl-2-pyrrolidone (NMP) and piperidine were obtained from Sigma-Aldrich^®^ and Thermofisher^®^. Solvents such as acetonitrile (ACN), methanol (MeOH), ethanol (EtOH), diethyl ether (Et_2_O), chloroform (CHCl_3_), dimethyl sulfoxide (DMSO), *N,N*-dimethylformamide (DMF), ethyl acetate (EtOAc), dichloromethane (DCM), acetic anhydride and hexane were used at synthesis grade purity and directly from commercial sources (Scharlab^®^, Panreac^®^ and VWR^®^). Trifluoroacetic acid (TFA) was purchased from Acros Organics^®^. The *N-*fluorenylmethoxycarbonyl (Fmoc)-protected amino acids (Fmoc-Arg (Pbf)-OH and Fmoc-Gly-OH), 2-(1*H*-Benzotriazole-1-yl)-1,1,3,3-tetramethyluronium hexafluorophosphate (HBTU) and the Rink amide MBHA (100–200 mesh) resin were obtained from Novabiochem^®^.

### Synthetic protocols

Ligand H_2_L1 and its corresponding complex C1 were re-synthesized and characterized as previously described (Peña et al., 2021). The synthesis of ligands H_2_L2-H_2_L5 and the corresponding copper(II) complexes (C2-C5) is described in the Supplementary Material S1.

Peptides (R_9_ and G_4_-R_9_) were synthesized using a microwave assisted Biotage^®^ Initiator + Alstra synthesizer, following standard Fmoc solid-phase peptide synthesis (SPPS) protocols (Chan & White, 2000). They were synthesized on a Rink amide MBHA resin (100–200 mesh) in a 0.25 mmol scale (0.59 mmol/g). The amino acids (4 eq) were assembled using HBTU (3.9 eq) as coupling agent, DIEA (8 eq in *N*-methyl-2-pyrrolidone (NMP)) as a base and DMF as a solvent. Fmoc deprotection was carried out at room temperature with 20% piperidine in DMF for about 20 min. Couplings were carried out at 75°C for Fmoc-Gly-OH (5 min). For Fmoc-Arg (Pbf)-OH residues, double couplings were carried out at 50°C (2 × 6.5 min). Cleavage and simultaneous removal of the protecting groups were done manually with a TFA/TIS/H_2_O (95/2.5/2.5, (v/v/v)) mixture for 2 h at room temperature. The resin was filtered out and washed with TFA. Filtrate and TFA washes were combined and TFA removed under a nitrogen stream. The final peptides were precipitated in cold Et_2_O, recovered by centrifugation, dissolved in water and lyophilized.

### High-performance liquid chromatography (HPLC)

Reversed-phase HPLC analyses were carried out on an Agilent Technologies 1,200 Series with a ultraviolet-visible (UV-vis) detector. A C12 Jupiter Proteo (90Å, 4 μm, 250 × 4.6 mm) and a C12 Jupiter Proteo Axia (90Å, 4 μm, 250 × 21.2 mm) columns were used for the analytical and preparative scales, respectively. A two-solvent gradient was used (*Solvent A*: H_2_O/TFA (99.9/0.1%); and *Solvent B* (ACN/H_2_O/TFA (90/9.9/0.1%)). Absorbance at 220, 260 and 310 nm were used to monitor the different compounds. Details of the gradients used are given in the corresponding synthetic procedures.

### Nuclear magnetic resonance (NMR) spectroscopy

NMR experiments were recorded on BRUKER DPX-250, 300, 360, 400 and 500 MHz instruments at the *Servei de Ressonància Magnètica Nuclear* (UAB) and Spectropole facilities (AMU). Deuterated solvents were directly purchased from commercial suppliers. All spectra have been registered at 298 K otherwise noticed. The abbreviations used to describe signal multiplicities are: s (singlet), d (doublet), dd (double doublet), t (triplet), bs (broad signal) and m (multiplet). All ^13^C NMR acquired spectra are proton decoupled.

### Mass spectrometry (MS)

Routine electrospray ionization (ESI)-MS measurements were recorded at the Spectropole facility (AMU) on a SYNAPT G2 HDMS (Waters) instrument with an ionization source at atmospheric pressure (API) pneumatically assisted and with a time-of-flight analyzer (TOF). Ionization conditions: electrospray voltage of 2.8 kV, capillary voltage of 20 V, dry gas flow at 100 L/h. High resolution (HR)-MS measurements were recorded after diluting the corresponding solid complexes in a MicroTOF-Q (Brucker Daltonics GmbH, Bremen, Germany) instrument equipped with an electrospray ionization source (ESI) in positive mode at the *Servei d’Anàlisi Química* (UAB). The nebulizer pressure was 1.5 Bar, the desolvation temperature was 180°C, dry gas at 6 L min^−1^, the capillary counter-electrode voltage was 5 kV and the quadrupole ion energy, 5.0 eV.

### Electron paramagnetic resonance (EPR) spectroscopy

EPR measurements were carried out on a BRUKER ELEXSYS 500 X-band CW-ESR spectrometer, equipped with a BVT 3000 digital temperature controller. The spectra were recorded at 120 K in frozen DMSO solutions otherwise noticed. Typical acquisition parameters were as follows: microwave power 10–20 mW, modulation frequency 100 kHz, modulation gain 3 G. Simulations were performed using the EasySpin toolbox developed for MATLAB (Stoll and Schweiger, 2006).

### Ultraviolet-visible (UV-vis) spectroscopy

All the spectra were recorded at room temperature either on an Agilent HP 8453, a Varian Cary 50 Bio, a Varian Cary 60 Bio or a Perkin Elmer Lambda 650 spectrophotometer, using 1 cm quart-cuvettes. *Ascorbate consumption experiments* were monitored by UV-vis at the maximum absorption band of the ascorbic acid (100 µM) at 265 nm for about 45 min. CuCl_2_ and the assayed complexes **C1**-**C3** were added at a final concentration of 2 µM in 50 mM NaCl/5 mM TRIS-HCl buffer (pH 7.2), with a maximum of 5% of DMSO.

### Inductively coupled plasma (ICP) optical emission spectrometry (OES) and mass spectrometry (MS)

ICP-MS was performed on an Agilent apparatus, model 7500ce. ICP-OES was carried out in a Perkin-Elmer, model Optima 4300DV. Both used standard acquisition parameters for copper content.

### Stock solutions of complexes C1-C5 for biological assays

Stock solutions of the assayed complexes C1-C5 were prepared by weighing the appropriate amount of complex and diluting them in the corresponding solvent (DMSO for C1, and H_2_O for C2-C5). Quantification of the copper concentration was carried out at the *Servei d’Anàlisi Química* (UAB) by ICP-OES. Measurements were performed at least per duplicate.

### Cell cultures

Human cancer cells (cervix: HeLa; and breast: MCF7) were obtained from American Type Culture Collection (ATCC, Manassas, VA, United States). Cells were routinely cultured with DMEM (Dulbecco’s modified Eagle’s medium, Invitrogen) for HeLa, and with DMEM/F-12 (Dulbecco’s MEM/Nutrient Mixture F-12 Ham, Invitrogen) for MCF7, both supplemented with 10% heat-inactivated fetal bovine serum (FBS, Invitrogen) at 37°C in a humidified 5% CO_2_ atmosphere.

### Cell-viability assays

HeLa and MFC7 cells were seeded into a 96-well plate at a cell density of 3·10^3^ cells/well in 100 µL of culture medium and allowed to grow overnight. The next day, the C1-C5 complexes were added to cells at a range concentration of 0–200 μM. Working concentrations of complexes C1-C5 (<0.1% DMSO in biological experiments, required for C1) were prepared in DMEM (for HeLa) and DMEM/F-12 (for MCF7) medium. The growth inhibitory effect of the complexes was measured at 24 h using the PrestoBlue Cell Reagent (Life Technologies) assay. Briefly, PrestoBlue (10 μL; resazurin-based solution) was added to each well. After 2 h incubation (37°C, 5% CO_2_), the fluorescence of each well was measured at 572 nm after excitation at 531 nm with a Microplate Reader Victor3 (Perkin Elmer). Cytotoxicity was evaluated by relative cell viability (%) for each sample related to the control well. The IC_50_ values were calculated from the obtained cell viability results. Each complex was tested per triplicate and averaged from three independent sets of experiments. Blank and complex controls were also considered. For experiments of C1 and C5 at 30 min and 4 h of treatment, cells were plated and treated following the same protocol. After each treatment time, the supernatant was removed, cells were washed, and fresh media was added. Cells were allowed then to evolve until a total incubation time of 72 h and the cell viability was measured with PrestoBlue^®^, as beforehand detailed.

### Cellular copper uptake studies

HeLa and MCF7 cells were plated, grown, and allowed to adhere overnight in a 6-well plate (2·10^5^ cells/well). Cells were treated for 4 h with the different copper complexes at the desired concentration. Before analysis, medium was removed, and cells were washed with DPBS (Dulbecco’s Phosphate Buffered Saline, Invitrogen) and trypsinized for 10 min. The samples were harvested by centrifugation (1,400 rpm, 5 min) and the cellular pellets were collected and digested with concentrated HNO_3_. Quantification of the intracellular copper was performed by using inductively coupled plasma mass spectrometer (ICP-MS). Measurements were performed at least per duplicate.

### Intracellular ROS production assays

HeLa and MCF7 cells were plated and allowed to adhere overnight in a 96-wells plate (2·10^4^ cells/well). The 2′,7′-dichlorofluorescin diacetate reagent (DCFDA, 25 μM in DMSO) was then added and the cells incubated at 37 °C in the dark for 30 min. The DCFDA solution was removed and cells were treated with C1 and C5 at the corresponding IC_50_ values (at 24 h) and incubated for 4 and 24 h. The experiments were run in triplicate. H_2_O_2_ was used as a positive control at 100 µM. The fluorescence of each well was measured at 535 nm with a Microplate Reader Victor3 (Perkin Elmer) after excitation at 485 nm.

### Antibody production and titer

Rabbit Poly-Arginine (9R) antibody was produced by Davids Biotechnologie GmbH (Germany) as follows. New Zealand white rabbits were immunized with newly synthesized Acetyl-RRRRRRRRR-Amide peptide and the antiserum was further purified by affinity purification. The obtained 9R antibody (Anti-R_9_) was tittered by means of ELISA (enzyme linked immunosorbent assay) to determine the optimal dilution for immunocytochemical detection. Briefly, MaxiSorp (Sigma-Aldrich, MO, United States) microtiter plate was coated with the Acetyl-RRRRRRRRR-Amide peptide in carbonate/bicarbonate buffer (pH 9.6) overnight at 4°C. The plate was washed three times with PBS-BSA 0.5% between every step. The remaining binding sites were blocked with PBS-BSA 0.5% for 2 h. The antiserum samples were serial diluted at a range concentration of 1/10–1/10,000 (v/v). The samples were applied in triplicate and incubated for 1 h at room temperature. HRP (horseradish peroxidase)-conjugated secondary antibody anti-rabbit (Bio-Rad) diluted in blocking buffer was incubated with the samples for 1 h at room temperature. TMB (3,3′,5,5′-tetramethylbenzidine, ThermoFisher) solution was added to each well and incubated for 30 min for signal detection. An equal volume of stopping solution (2 M H_2_SO_4_) was added and the completed reaction was read at 450 nm in a microplate reader (Tecan).

### Immunofluorescence assays

HeLa cells were seeded in 24-well plates at a cell density of 5·10^4^ cells/well in 1 ml of culture medium and allowed to grow overnight on a sterilized coverslip. The next day, cells were incubated with 50 µM of C5 or control peptide (R_9_) for 4 h. For cell fixation, culture medium was removed, cells were washed with DPBS and fixed with 4% paraformaldehyde (PFA, Sigma-Aldrich) at room temperature for 20 min. PFA was then removed and cells were washed and blocked with PBS containing 3% BSA (bovine serum albumin, Sigma-Aldrich) and 0.1% Triton X-100 (Sigma-Aldrich) for 30 min. Cells were then incubated overnight at 4 °C with Anti-R_9_ antibody (Rabbit, 3.4 μg/ml) and anti-LAMP-1 (Rat, 2 μg/ml, Santa Cruz Biotechnology, CA, United States). Polyclonal IgG anti-rabbit Alexa 568, and anti-rat Alexa 488 antibody (1:500, Thermo Scientific) were added after removal of the primary antibodies and washing cells three times with PBS (1% BSA and 0.1% Triton X-100). Nuclei and cell membrane were counterstained with 4’,6-diamidino-2-phenylindole (DAPI) (1:500, 5 min) and Alexa-633 conjugate of wheat germ agglutinin (WGA) (2 μg/ml, 10 min, Thermo Scientific) respectively. The slides were mounted and prepared for fluorescence and confocal microscopy. Images were captured using a Leica TCS SP5 laser scanning confocal microscope with a PL APO 63×/1.4-0.6 Oil objective and processed using Imaris software.

### Statistical analysis

Results are presented as means ± standard deviation in all figures and in Table 2. Each viability assay determination experiment was performed in three independent experiments, while each confocal microscopy and cellular uptake experiment was performed in at least two independent experiments. GraphPad Prism was used for graphs and associated calculations.

## Results

### Synthesis and characterization of the ligands H_2_L2-H_2_L5 and the corresponding copper(II) complexes

**SCHEME 1:**
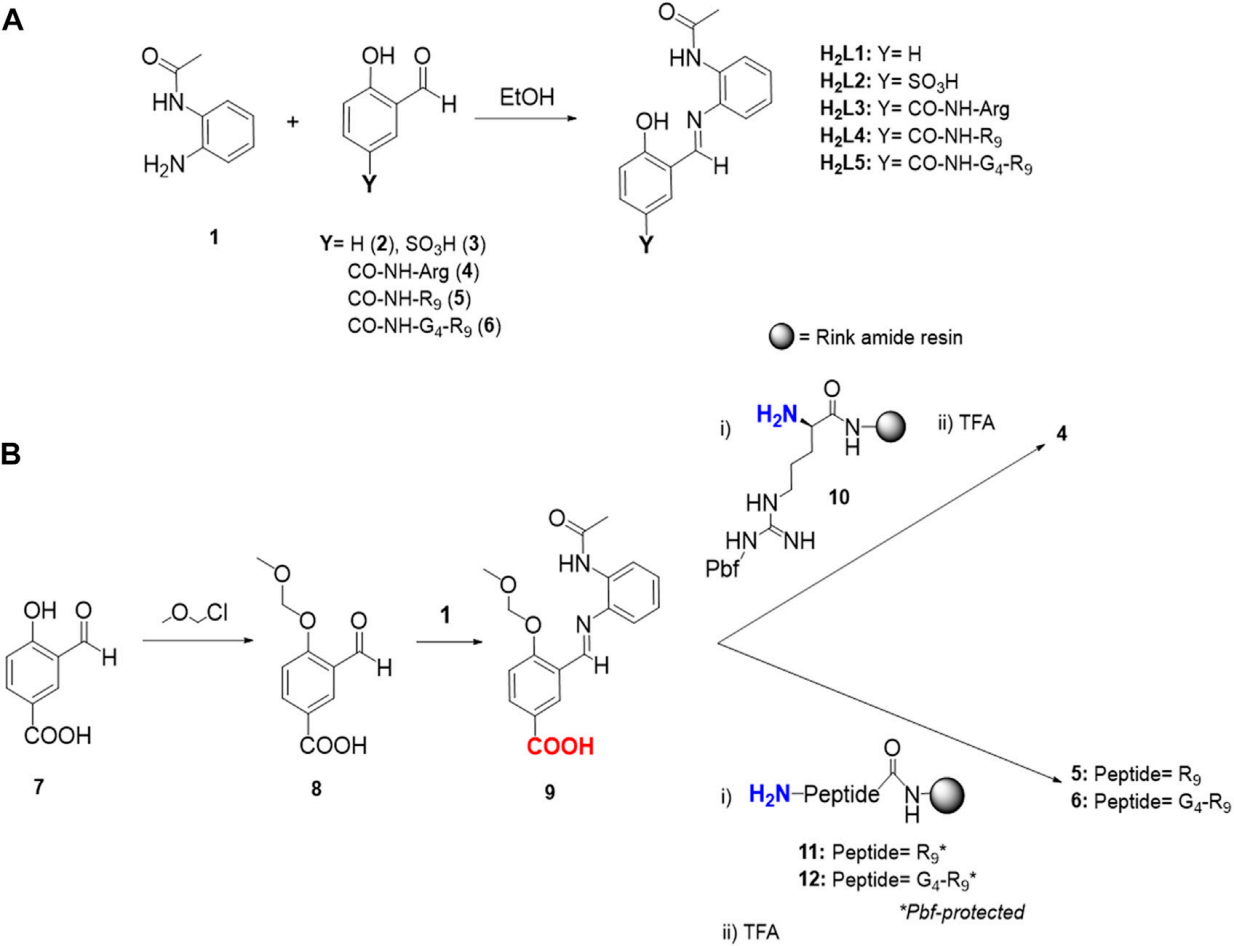
Synthetic procedures to obtain H_2_L1 functionalized ligands (H_2_L2-H_2_L5). **(A)** Condensation reaction to obtain ligands H_2_L2-H_2_L5 from the corresponding precursors 3-6. Arginine in 4, peptide sequences in 5 and 6, and H_2_L3-H_2_L5 have an amide group instead of a carboxylic group at the C-terminal site. **(B)** Schematic synthetic procedure to obtain salicylaldehyde functionalized precursors 4-6. Arginine side-chain in peptides 11 and 12 (R_9_*) have a 2,2,4,6,7-pentamethyldihydrobenzofuran-5-sulfonyl (Pbf) protecting group (PG). Colored in blue, the amine terminal functional group employed to form the amide bond with the carboxylic acid (-COOH, in red) following standard HBTU coupling procedure. TFA refers to trifluoroacetic acid.

Ligand H_2_L1 and its corresponding complex C1 were synthesized as previously reported (Peña et al., 2021). The four newly synthesized ligands H_2_L2-H_2_L5 were essentially prepared by imine bond formation between the monoprotected benzene-1,2-diamine (1) and the corresponding salicylaldehyde precursor (**3-6**, Scheme 1A). The synthesis of the different ligands and their respective precursors, including the corresponding characterization data, can be found in the experimental section and as part of the Supplementary Material (Supplementary Figures S1-9).

For H_2_L2, aldehyde (3) was achieved through a *para*-sulfonation of the starting material 2*-*hydroxybenzaldehyde (2), via an electrophilic aromatic substitution (S_E_Ar) reaction (Clayden et al., 2012). Standard sulfonation reactions are normally performed at temperatures around 100°C, however, this step was carried out at 40°C to prevent significant oxidation of the aldehyde in such acidic conditions (Kirker, 1980). Regarding the synthesis of the salicylaldehyde precursors of the ligands H_2_L3-H_2_L5 (compounds 4–6), similar procedures were followed for all three, namely attaching the Arg residue (H_2_L3, precursor 4) or the corresponding CPPs (H_2_L4-H_2_L5, precursors 5 and 6, respectively) to the common intermediate 9 through a stable amide linkage *via* standard solid-phase Fmoc-methodologies (Scheme 1B) (Chan and White, 2000).

A Rink amide resin was chosen in all the cases to obtain an amide functional group in the *C*-terminal position of the Arg or CPP scaffold, in order to avoid competition in the Cu(II) complexation step (*vide infra*). Deprotection and cleavage steps from the resin were performed using standard methods, and preparative reversed-phase HPLC purification rendered the pure salicylaldehyde precursors 4-6. To note, the optimized strategy to synthesize the precursors 4-6 required the use of the imine intermediate benzoic acid 9, which was obtained by condensation reaction between the MOM-hydroxyl protected compound 8 and the monoprotected diamine 1. The phenol protection with the MOM-group and the imine-bond formation (the latter as a strategy to mask the aldehyde group) were crucial to increase the yield of the coupling reactions between 9 and 10-12, and to avoid the formation of by-products.

The Cu(II) complexes C2-C5 were obtained after metalation of the respective H_2_L2-H_2_L5 ligands with copper(II) acetate following the same procedure as previously described for C1 (Peña et al., 2021). The complexes were isolated from the reaction media by precipitation. Experimental evidence of Cu(II) complexation was initially obtained for the complexes with the two simplest modifications (i.e., C2 and C3) by the appearance of metal-to-ligand or ligand-to-metal charge transfer bands (MLCT, λ∼ 420 nm), and Cu(II) *d*-*d* bands (λ∼ 650 nm) in the ultraviolet-visible (UV-vis) spectra (Supplementary Figure S10). Additionally, the electronic transitions observed in the functionalized complexes are analogous to those obtained for C1, outlining that the chemical derivatization did not affect the coordination capabilities of the ligands. The presence of the molecular peaks for C2-C5 in high-resolution mass spectrometry (HR-MS, Supplementary Figure S11) corroborated the successful metal coordination in all cases.

**FIGURE 2:**
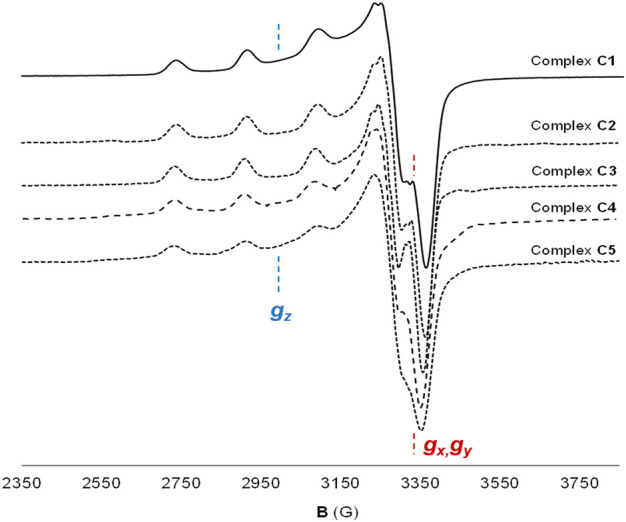
Cu(II) coordination environment of complexes C1-C5. X-EPR band spectra of complexes C1-C5 in frozen DMSO solution at 120 K, confirming the same Cu(II) coordination environment in all the complexes.

Comparison of the EPR spectroscopic features and calculated characteristic parameters (*g* and *A* tensors) of C2-C5 with those of the parent complex C1 (Figure 2; Table 1) confirmed that the newly synthesized complexes do maintain the same Cu(II) coordination environment as C1, with the metal center located in a N_2_O_2_ in-plane coordination environment (Peisach and Blumberg, 1974) and with the unpaired electron in a $d_{x^{2}-y^{2}}$ orbital (*g*_z_ > *g*_x,y_ > *g*_e_), thus adopting a square-planar or square-pyramidal derived geometry. The calculated *g*_z_/*A*_z_ ratio is lower than 140 cm for all of them, which is indicative of a non- or marginally-distorted structure from planarity (Sakaguchi and Addison, 1979). Consequently, and taking all the above insights together, the different functionalization strategies assayed have not altered the metallic core of C1, present in all new complexes C2-C5.

**TABLE 1:** EPR parameters from X-EPR band spectra of complexes C1-C5. Spectra were recorded at 120 K in frozen DMSO solutions. g_z_/A_z_ ratio is a parameter that predicts the distortion from planarity of the structure (g_z_/A_z_ > 140 cm to consider a planar distortion in the structure).

Complex	g_z_	A_z_ (10^−4^ cm^−1^)	g_x/_g_y_	A_x_ A_y_ (10^−4^ cm^−1^)	g_z_/A_z_ (cm)
C1	2.244	183	2.043/2.076	<30	123
C2	2.246	183	2.040/2.074	<30	123
C3	2.247	184	2.045/2.071	<30	122
C4	2.247	185	2.041/2.074	<30	121
C5	2.247	185	2.047/2.075	<30	121

### Effect of functionalization on the copper-mediated ROS production capabilities

Prior to biological evaluation, and after proving that the new C2-C5 Cu(II) complexes hold the same metallic core as C1, the impact of the ligand substituents on their catalytic properties, and therefore, ROS-generating capabilities (Peña et al., 2021), were confirmed with the two simplest functionalized Cu(II) compounds (C2 and C3), as model compounds bearing oppositely-charged functional moieties. The consumption of ascorbate was measured by UV-vis spectroscopy, monitoring its characteristic absorbance at 265 nm. The correlation of ascorbate consumption with the generation of ROS has proven to be a reliable method to effectively monitor ROS production (Alies et al., 2013; Chassaing et al., 2013).

**FIGURE 3:**
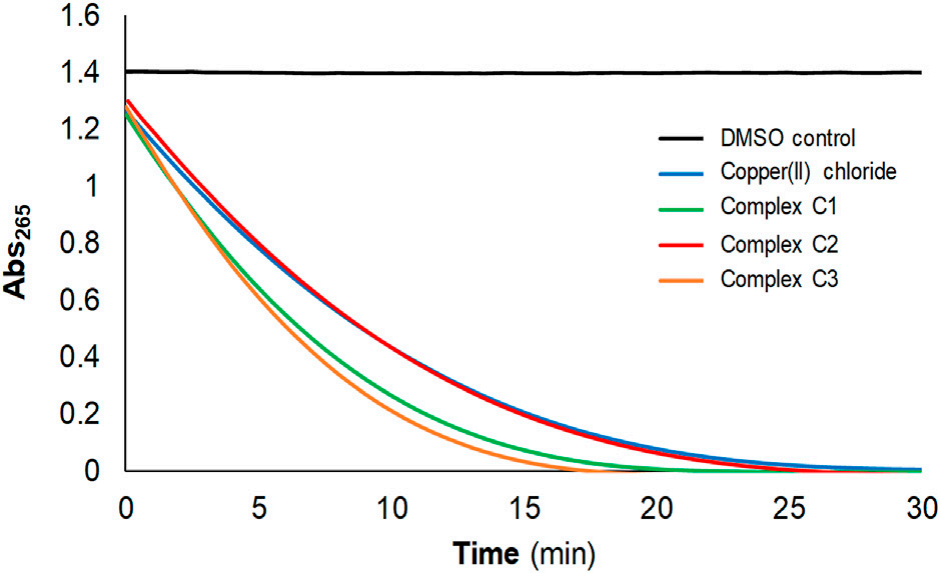
Confirmation of reactive oxygen species (ROS)-generation capabilities after functionalization using the ascorbate consumption experiment. Ascorbic acid (100 µM) consumption of CuCl_2_, C1, C2 and C3 (2 µM) monitored by UV-vis spectroscopy at 265 nm, in 50 mM NaCl/5 mM TRIS-HCl buffer at pH 7.2.

In the presence of ascorbate and in an aerobic environment, Cu(II) catalyzes the generation of ROS upon ascorbate consumption (Halliwell and Gutteridge, 1990). Without copper (DMSO control), no decrease in the absorbance at 265 nm was observed (Figure 3). In contrast, free Cu(II) ions (CuCl_2_) produced a rapid decrease in the absorbance and, after 20 min, the ascorbate was almost totally consumed. For the sake of comparison, the ascorbate consumption capabilities of **C1**, **C2** and **C3** were examined at the same time and conditions. C3 consumed ascorbate at similar rates to C1, and just slightly slower rates were observed for C2 (Figure 3), with total consumption after 25 min in all cases.

### Cytotoxicity assays and intracellular copper(II) accumulation

**FIGURE 4:**
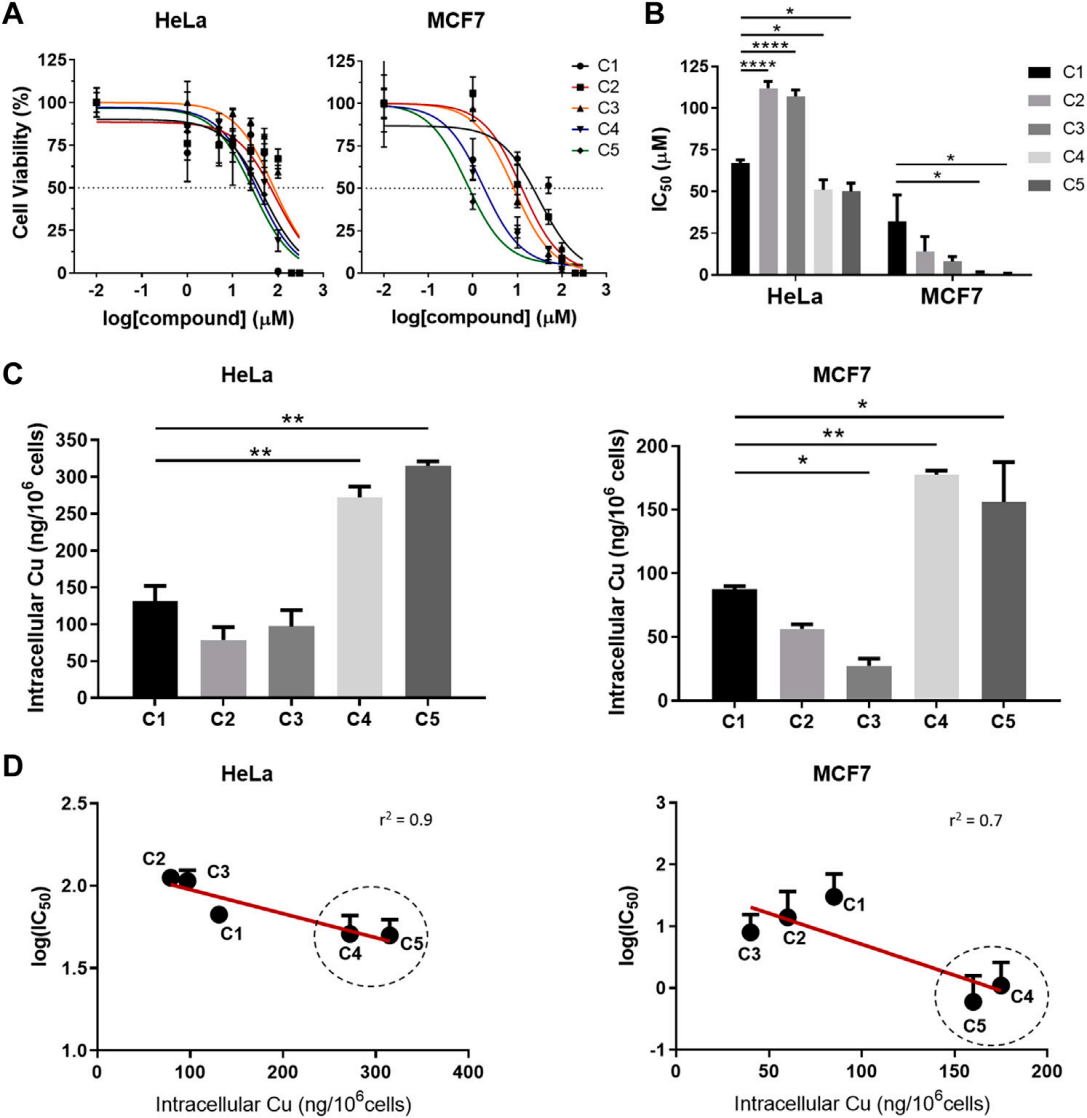
*In vitro* cytotoxicity and cellular internalization of C1-C5 in HeLa and MCF7 cancer cells. **(A)** Cell-viability assays for complexes C1-C5 in HeLa and MCF7 cancer cell lines after 24 h treatment. **(B)** IC_50_ values for complexes C1-C5 after 24 h treatment, calculated from the corresponding cell-viability curves fitting (Figure 4A). **(C)** Quantification of the copper (Cu) uptake in HeLa and MCF7 cell lines after 4 h treatment with complexes C1-C5 (50 µM). **(D)** Correlation between the cytotoxicity (log (IC_50_)) and the Cu uptake for HeLa and MCF7, with the cell-penetrating peptide-bearing Cu(II) complexes circled. Concentrations of the complexes are normalized in all cases based on Cu concentration for comparison purposes. Statistical differences were determined using a one-way ANOVA test, with *p* values as: without symbol (*p* > 0.05, non-significant), *p ≤ 0.05, **p ≤ 0.01, ***p ≤ 0.001, ****p ≤ 0.0001. Results average at least three independent experiments (N ≥ 3) for IC_50_ values, and two (N ≥ 2) for the Cu uptake. Statistical analysis and goodness-of-fit for linear regression (*r* ^2^) using alpha threshold of 0.05 were calculated using GraphPadPrism.

*In vitro* assays with the complexes C2-C5 were carried out in HeLa and MCF7 cancer cells in order to (1) compare their activity with that of C1 (Figure 4), which we previously reported to be cytotoxic against several cell lines (Peña et al., 2021), and to (2) elucidate the effect of the different derivatizations. All the C2-C5 synthesized complexes are soluble in water (at least 5 mg/ml for **C2**-**C3**, and 20 mg/ml for **C4**-**C5**) and in biological medium, without the need of any percentage of DMSO (as it was required for the biological evaluation of C1 because of solubility issues). The dose-response curves are shown in Figure 4A, and the obtained IC_50_ values are plotted in Figure 4B and summarized in Table 2. To ensure comparability, all the stock solutions of C1-C5 were standardized by ICP-MS prior to biological evaluation and results normalized based on the copper content.

**TABLE 2:** IC_50_ (µM) values at 24 h of complexes C1-C5 in HeLa and MCF7 cell cultures, normalized for copper (Cu) concentration. The results shown are representative of at least three independent experiments (N ≥ 3).

Compound	HeLa	MCF7
C1	67 ± 2	32 ± 16
C2	112 ± 4	14 ± 9
C3	107 ± 7	8 ± 3
C4	51 ± 6	1.1 ± 0.6
C5	50 ± 5	0.6 ± 0.4
CuCl_2_	≥ 200	≥ 200

As beforehand mentioned, the first derivatization strategy (sulfonate and Arg) mainly aimed at increasing the solubility of the C2 and C3 complexes respect to C1. Results underline that, although improved aqueous solubility was attained, none of the two modifications contribute to enhance the cytotoxicity of the parent complex in cancer cells, with IC_50_ values higher than (in HeLa), and in the range of (in MCF7) complex C1 (Figure 4B). In contrast, functionalization with the CPPs (complexes **C4** and **C5**) resulted in improved cytotoxicity with significantly lower IC_50_ values, particularly in MCF7 (IC_50,**C1**_-to-IC_50,**C4-C5**_ ratio ≥30, Table 2), thereby suggesting that the intracellular delivery plays a key role in the final activity.

Thus, cellular uptake studies were carried out to deepen into the impact of the tailored modifications on the internalization of the five complexes C1-C5. ICP-MS was used to quantify the amount of Cu(II) inside cells (Figure 4C). Intracellular copper content after treatment with complexes C1-C3 was below 150 ng/10^6^ cells, with similar uptake values among the three. Importantly, while sulfonate and Arg derivatizations, with the concomitant increase in solubility of the corresponding C2 and C3 complexes, did not mediate copper uptake, functionalization with CPPs (C4 and C5) significantly promoted intracellular copper delivery (Figure 4C). The amount of metal able to accumulate inside cells for the CPP-conjugated complexes is at least about twofold the amount observed for C1 (272 ± 15 and 315 ± 6 versus 131 ± 21 ng/10^6^ cells in HeLa, and 178 ± 2 and 156 ± 22 versus 88 ± 2 ng/10^6^ cells in MCF7, for C4 and C5 versus C1, respectively). Based on the data, the presence of the G_4_ linker in C5, which was designed to provide higher conformational flexibility (and ideally better interaction of the peptide with the cell membrane), did not result in clear differences in uptake or cytotoxicity as compared to C4 (at least in the assayed cancer cell lines thus far).

Although there are multiple factors influencing the (re)activity of metal complexes (both from chemical and biological features), we attempted to evaluate how strongly the intracellular delivery is related to the anticancer activity of these compounds. Thus, we correlated the obtained IC_50_ values (in *log*) with the copper uptake (intracellular copper amount). As exemplified in Figure 4D, this resulted in a very good correlation for HeLa cancer cells (with *r* ^2^ = 0.9 and *p* value of 0.02). For MCF7, the correlation was still fairly good (*r* ^2^ = 0.7) and, despite not being strictly significant by definition (*p* value of 0.08), there is a clear tendency outlining intracellular copper delivery as an important design parameter to modulate cytotoxicity in these (and, by extension, likely in similar) Cu(II) complexes.

### Effect of cell-penetrating peptide-conjugation: Reactive oxygen species, efficiency and cellular distribution

**FIGURE 5:**
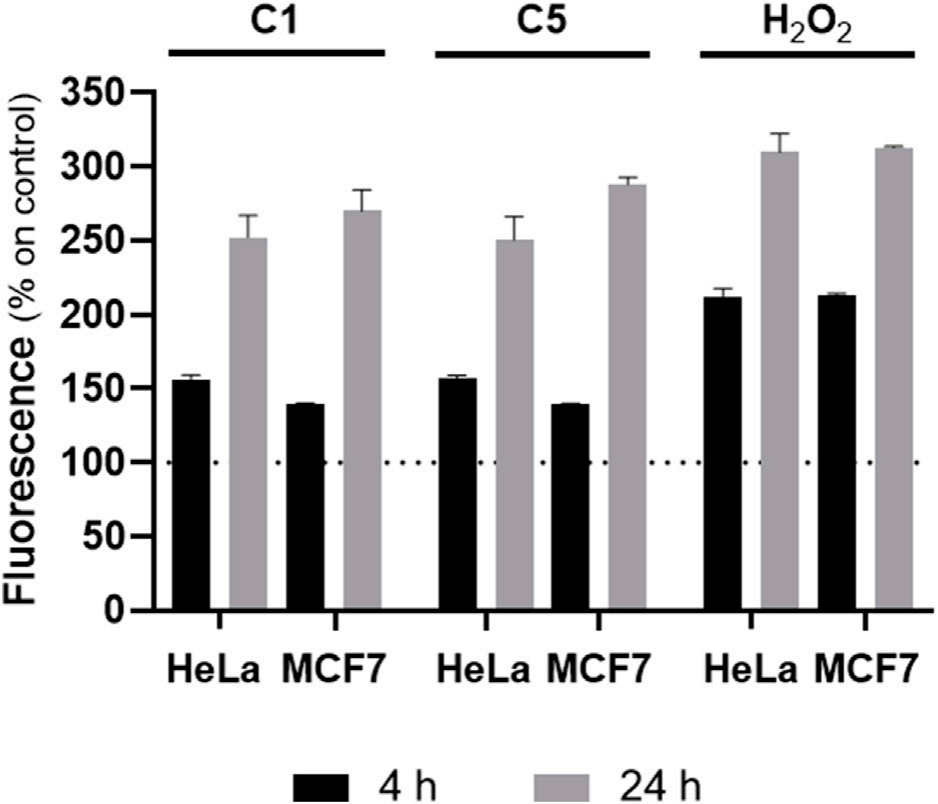
*In vitro* reactive oxygen species (ROS) production. Intracellular levels of ROS, measured with the DCFDA assay in HeLa and MCF7 cancer cells for complex **C1** and **C5** at their IC_50, 24h_ values and two different times (4 and 24 h). H_2_O_2_ (100 µM) was used as positive control. The values are plotted in percentage respect to cells control, which is 100%.

To gain more insights into the behavior of the CPP-bearing Cu(II) complexes, and taking into account the similar profile exhibited by **C4** and **C5** (with slightly higher toxicity for the latter), **C5** was selected for further comparative evaluation with **C1**. We initially corroborated the *in vitro* ROS production capabilities of **C5** using the DCFDA assay (Figure 5). Data confirm that the CPP-conjugated Cu(II) complex at their IC_50_ value was successfully able to produce significant levels of ROS in both tested cancer cell lines, reaching similar values to the positive control H_2_O_2_ (especially after 24 h incubation) and, thus, reinforcing the ROS-mediated pathway as an important mechanism of action for this set of complexes. It is also worth mentioning that, especially in MCF7 cancer cells, complex C5 was able to trigger the generation of a similar level of ROS to C1 yet using 50-fold less amount of complex, in line with the observed enhanced uptake and higher cytotoxicity of C5.

**FIGURE 6:**
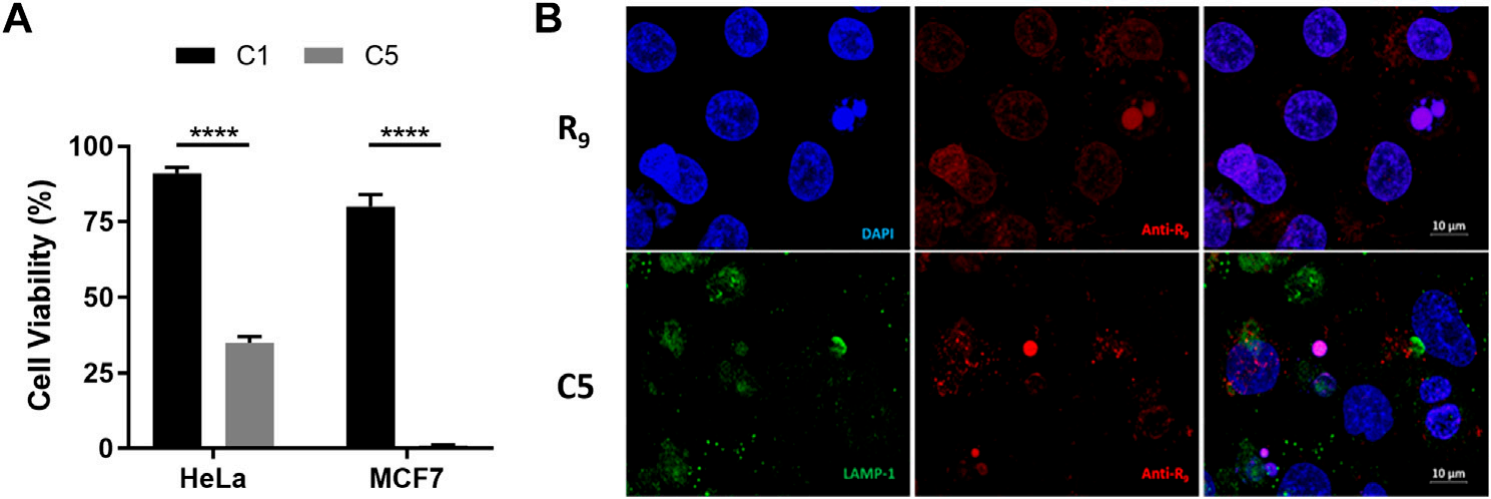
Cellular localization and cytotoxicity efficacy of cell-penetrating peptide-conjugated Cu(II) complex C5. **(A)** Cell-viability assays for complexes C1 and C5 (100 µM) in HeLa and MCF7 cell lines at 30 min or 4 h treatment times, after which the cell media was replaced by fresh medium and cells incubated for extra time until reaching a total of 72 h. Statistical differences were determined using a one-way ANOVA, with *p* values as: ****p ≤ 0.0001. **(B)** Cellular distribution of R_9_ peptide (control experiment) and complex **C5** after 4 h incubation in HeLa cancer cells. HeLa cells were fixed and stained with Anti-R_9_ (red) and anti-LAMP-1 (green, for lysosomes) antibodies, and DAPI (blue, nuclei). Last column shows the merged images.

We then set out to evaluate the impact of the CPP-mediated internalization in the cytotoxic efficacy of the final complex. To this end, cytotoxicity assays were performed at shorter treatment time for C1 and C5 (4 h, Figure 6A). After that specific incubation time, the supernatant of the cells was removed (containing the non-internalized complex), cells were washed, new cell culture medium added, and cells were incubated to complete 72 h of total incubation time. Data show that whilst there is almost no cell-death for C1, C5 drastically reduced the cell viability to more than 60% in HeLa and almost 100% in MCF7 after 4 h treatment (Figure 6A). These results reveal that the presence of the CPP not only promoted intracellular delivery, but, consequently, it also mediated a faster anticancer response, understood as a significantly higher activity than the parent complex C1 at short incubation times. The increased cytotoxicity-to-time ratio observed for C5 would contribute to enhance therapeutic efficiency, also potentially minimizing the extent and impact of clearance and drug resistance pathways of tumoral cells due to the faster action.

Finally, we studied the subcellular localization of C5 in comparison to the CPP moiety alone in order to elucidate the fate of the conjugated cargo inside the cells. Several biophysical methods have been traditionally used to assess the cellular distribution of CPPs and their cargoes (e.g., fluorescence, radiolabeling, Raman spectroscopy and X-Ray scattering), being those based on fluorescence the most extensively used (Bechara and Sagan, 2013). Usually, peptides are covalently coupled to a fluorophore, and monitoring of the fluorescence allows for cellular localization with confocal microscopy. However, besides all the experimental factors that may influence the uptake mechanism and subsequent localization (e.g., cell type, incubation time and temperature), the functionalization of CPPs with dyes has been reported to alter their inherent cellular distribution. This is basically because fluorophores are generally hydrophobic and they can change the solubility, flexibility and conformation of the final CPP functionalized complexes (Szeto et al., 2005; Puckett and Barton, 2009; Mishra et al., 2011; Hirose et al., 2012). Thus, in order to avoid interference or misleading conclusions, we used immunofluorescence with an Anti-R_9_ antibody (specifically produced with a R_9_ peptide as antigen; Acetyl-R_9_) to provide an initial proof-of-concept of the intracellular distribution of complex **C5** in HeLa cells (Figure 6B).

Analysis with confocal microscopy after 4 h incubation time revealed a diffused distribution throughout the cytosol compartment, with few punctual accumulations in the nuclei. These results demonstrate that C5 was able to accumulate inside cells after 4 h, which is in concordance with what was observed in the copper uptake experiments (Figure 4C). In contrast, incubation with the Arg-rich CPP (R_9_ peptide, Figure 6B), as a control, showed localization in both the cytoplasm and the nuclei, with preferential accumulation in the nuclei, where it remains most-likely retained partly due to the interaction with the negatively-charged phosphate backbone of the DNA. In order to elucidate whether the linker modulates subcellular localization, we then also carried out immunofluorescence assays with C4. As observed from the confocal microscopy images (Supplementary Figure S12), the intracellular distribution of C4 was analog to that of C5 (i.e., mostly diffusively localized in the cytosol), thus overall suggesting that the linker does not have a clear impact on intracellular distribution either. Promisingly, both C4 and C5 results show no colocalization of the CPP-conjugates in the lysosomes after 4 h incubation which, together with the diffused distribution pattern in the cytosol, could indicate potential endo-/lysosomal bypass and/or escape.

## Discussion

Metals and their corresponding complexes have already shown remarkable contributions in oncology, both in diagnosis (e.g., gadolinium or technetium) and therapy (e.g., Pt) (Mjos and Orvig, 2014; Anthony et al., 2020). Despite the significant side-effects encountered, the three worldwide approved Pt-drugs (cisplatin, carboplatin and oxaliplatin) are still administered to at least 20% of cancer patients nowadays, and they represent first-line treatments for testicular, ovarian, bladder and colorectal cancer, among others, thus underlining the (still fully unlocked) potential of metal-based anticancer therapy (Casini et al., 2019; Rottenberg et al., 2021; Peña et al., 2022). The recent years have evidenced that complexes based on physiological (catalytic) metals that act *via* different mechanisms of action, like ROS generation, can offer promising therapeutic responses (Li et al., 2020; Liu et al., 2019; Lovejoy et al., 2011; Luo et al., 2014; Shen et al., 2020; Trachootham et al., 2009; Zhang and Sadler, 2017). Additionally, several studies emphasize the relevance of ROS-based cancer therapy as one of the strategies to avoid and/or bypass Pt-induced resistance while increasing therapeutic selectivity towards cancer cells (Cui et al., 2018; Wang et al., 2021).

In this context, Cu(II) complexes have exhibited potential as anticancer agents (Santini et al., 2014). They can combine both features: they encompass a (more) biocompatible metal center and the possibility of triggering ROS production, as we and others have recently reported (Zubair et al., 2013; Ng et al., 2014; Sîrbu et al., 2017; Peña et al., 2019, 2021). However, certain physicochemical properties can limit the anticancer activity and hamper further (pre)clinical evaluation of newly developed (metal) anticancer agents. One crucial aspect relies on the lack of adequate solubility in biological fluids (Savjani et al., 2012) (about 90% of preclinical drug candidates present low-water solubility (Kalepu and Nekkanti, 2015)), as was for instance observed for our recently reported ROS-producing cytotoxic Cu(II) complex **C1** (Peña et al., 2021). A second key parameter in (metallo)drug discovery is based on the capacity to cross (cellular) membranes (which is related to properties such as lipophilicity) (Vargason et al., 2021).

Over the years medicinal chemistry has explored and established multiple strategies to overcome such limitations and efficiently deliver chemotherapeutics; many of them involving structural modifications like adding pH-sensitive groups that enhance aqueous solubility and conjugation to CPPs, among others. CPPs are typically made up of 5–30 amino acids, and they can be utilized as molecular transporters to facilitate the passage of therapeutic drugs across physiological barriers (Copolovici et al., 2014; Guidotti et al., 2017). Up to now, (cationic Arg-rich) CPPs have been widely used in many anticancer treatment strategies, successfully addressing both solubility and intracellular delivery issues of a variety of anticancer therapeutic molecules (Zhou et al., 2022).

Our goal in this work was to improve the anticancer activity of the promising ROS-producing Cu(II) complex C1 by employing solubility- and/or uptake-targeting ligand modifications like CPP-conjugation. However, there is still a lack of rationalization and understanding of how structural features and chemical modifications can influence the activity of the final compound, especially in metal-containing systems. Different from purely organic-based compounds, the (re)activity of metal-based structures is not only dependent on the metal center (and its oxidation state) or on the ligand scaffold, but also (and arguably even more) on the metal-ligand interaction (Anthony et al., 2020; Peña et al., 2022). Consequently, systematically and comparatively exploring several of such relevant and widely used derivatizations, especially CPP-conjugation, enabled us to deepen, at the same time, into structure-behavior relationships of Cu-based anticancer agents from different aspects, mostly encompassing physicochemical properties like solubility, (*in vitro*) ROS-generation capabilities, cytotoxicity, intracellular metal delivery and subcellular distribution.

For that purpose, ligand H_2_L1 was functionalized with a sulfonate group (H_2_L2) and an Arg residue (H_2_L3), with the main goal of increasing solubility, and with two variants of a nona-Arg CPP (Guidotti et al., 2017) (without linker, H_2_L4, and with, H_2_L5), for both solubility and intracellular delivery. Our data confirmed that the ligand scaffold was successfully tailored with the different functionalities, being the two variants of CPPs (for H_2_L4 and H_2_L5) linked through an analog chemical procedure as that carried out for the single Arg residue (H_2_L3). Noteworthy, conjugating Arg and Arg-rich CPPs to the ligand scaffold *via* the same chemical protocols outlines the robustness and versatility of such derivatization approach, which can be then analogously extended to future tailoring of this and similar (salphen-based) ligands and metal complexes with other peptides or cancer-targeting moieties containing amine functional groups. All the resulting Cu(II) complexes (C2-C5) exhibited improved water solubility.

Given that the cytotoxic activity of the Cu(II) complex C1 is strongly linked to ROS production (Peña et al., 2021), we specifically designed the functionalization strategies to be located on the periphery positions of the ligand H_2_L1, distant to the Cu(II) coordinating atoms to minimize alterations of the metallic core and preserve its ROS generation capabilities. Cytotoxicity assays in HeLa and MCF7 cancer cells highlighted that while the functionalization with sulfonate (C2) or Arg (C3) groups (i.e., modifications only targeting solubility issues) did not represent any improvement in the final *in vitro* anticancer activity, the conjugation to CPPs (i.e., complexes C4 and C5) promoted higher, faster and, therefore, enhanced ROS-mediated anticancer activity respect to C1; overall in line with the increase in intracellular copper levels. The differences observed in copper uptake can be thus directly attributed to the presence of the CPPs, which actively mediate the cellular internalization of C4 and C5 and, correspondingly, impact on their cytotoxicity.

Importantly, we confirmed that the presence of only one Arg residue (C3) is not sufficient to enhance cellular uptake despite improving aqueous solubility and, by extension, bioavailability. In some cases, it even represented a drawback regarding intracellular copper delivery and cytotoxicity (e.g., in HeLa cancer cells). These results highlight that specific solubility-targeting derivatizations do not correlate with an enhanced anticancer activity, and that they might indeed negatively impact other key drug properties such as cellular membrane penetrating capacity. All in all, the conjugation with (Arg-rich) CPPs can target and maximize both properties at once, thus underlining the importance of the adequate choice of chemical modifications in a more rational and holistic manner. Further systematic evaluation of these (and similar CPP-conjugated) metal complexes regarding biocompatibility and therapeutic performance in healthy primary cells and *in vivo* models would provide further insights into the added value of CPP-conjugation strategies in the development of metal-based anticancer agents.

Finally, we set out to explore the subcellular distribution of the CPP-conjugates as compared to the free peptide. For both C4 (without linker) and C5 (with linker), the ubiquitous intracellular distribution in the cytosol, together with the absence of specific lysosomal co-localization, might suggest non-endosomal uptake or rapid endosomal entrapment followed by endosomal release (Guterstam et al., 2009; Liu et al., 2018). One of the commonly reported intracellular delivery pathways of cell-penetrating peptides relies on endocytosis-like mechanisms. This generally results in the entrapment of the peptides and their cargo, (i.e., the Cu(II) complexes), inside the endosome and, subsequently, lysosomal compartments, whose low pH (4–5) and high enzymatic activity can degrade the compound and, hence, impair its activity (Lecher et al., 2017; Pei and Buyanova, 2019; Kondow-Mcconaghy et al., 2020). Nonetheless, in other cases (e.g., for specific sequences of Arg-rich CPPs, certain cargoes, type of cell and at determined concentrations), other mechanisms involving (passive) direct translocation or fusion pore formation have also been relevantly proposed (Brock, 2014; Allolio et al., 2018). Although it is still complex to univocally elucidate the underlying entry pathways for CPP-conjugates, the comparison of the different distribution profiles between the R9 peptide alone and both C4 and C5 corroborates that the attachment of the CPP to the Cu(II) complex has altered the intracellular biodistribution pattern. Additionally, the overall data suggest that the presence of the linker has no apparent effect in the intracellular distribution either, in line with the similar cytotoxicity and intracellular copper levels observed for both C4 and C5. It is undeniable that further elucidation of the molecular details of the entry and endosomal escape pathways, especially when involving metal-based compounds, are required to strengthen (metallodrug) structure-function relationships (Brock, 2014).

In conclusion, our data suggest that intracellular copper delivery plays an important role in governing the final cytotoxicity of redox-active Cu(II) complexes, while only tuning solubility by adding (positively/negatively) charged groups does not translate into an increased cytotoxic activity, and may also hinder intracellular delivery and hamper crossing (cellular) membranes. Beyond the therapeutic potential shown by the CPPs-conjugated complexes (C4 and C5), we expect that the systematic evaluation and understanding about the role of the different tailored modifications contributes to (1) enhance the value of (Arg-rich) CPP-conjugation in (metallo)drug discovery, and to (2) optimize the design of these and similar (redox-active) metal complexes for enhanced and faster intracellular delivery and, consequently, improved anticancer activity.

## Data Availability

The original contributions presented in the study are included in the article/Supplementary Material, further inquiries can be directed to the corresponding authors.
