# Supplementary material for: Cell-penetrating peptide-conjugated copper complexes for redox-mediated anticancer therapy
Source: Front Pharmacol. 2022 Nov 15;13:1060827. doi: 10.3389/fphar.2022.1060827 (PMC9714576; doi:10.3389/fphar.2022.1060827)
Supplement: Supplementary file 1 [file DataSheet1.docx]

**Supplementary Material**

1. **Synthesis of ligands H_2_L2-H_2_L5 and copper(II) complexes**

***N*-(2-aminophenyl)acetamide (1)** (Shirin et al., 2002). Acetic anhydride (5.12 mL, 51.2 mmol, 1 eq) was added dropwise at 0 °C under N_2_ atmosphere to a solution of benzene-1,2-diamine (5.54 g, 51.2 mmol, 1 eq) in anhydrous DCM (75 mL). The mixture was stirred for 2 h at 0 °C and then stored at -35 ºC overnight. The precipitate was filtered off and washed with cold DCM (3x5 mL) and diethyl ether (3x5 mL) to yield 1.01 g of a white solid. The filtrate was furtherly concentrated to the half of its volume and stored at -35 °C for 48 h more. The new precipitate was filtered off to render additional 1.56 g of product. Yield: 38 % (2.57 g). ^1^H NMR (360 MHz, *d_6_*-DMSO): δ 9.12 (s, 1H), 7.14 (d, *J* = 7.8 Hz, 1H), 6.88 (t, *J* = 7.4 Hz, 1H), 6.70 (d, *J* = 8.0 Hz, 1H), 6.52 (t, *J* = 7.3 Hz, 1H), 4.85 (s, 2H), 2.03 (s, 3H).

**3-formyl-4-hydroxybenzenesulfonic acid (3)** (Kirker, 1980). 2-Hydroxybenzaldehyde (600 mg, 4.90 mmol, 1 eq) was added dropwise into 5 mL of concentrated sulfuric acid at 0 °C. The reaction was stirred for 24 h at 40-45 °C. The reaction was cooled down to room temperature and poured into 20 mL of ice. Sodium carbonate (5 g, 47.17 mmol, 10 eq) was slowly added at 0 °C. The mixture was stirred at room temperature for 2 hours. The carnation precipitate was filtered off and washed with EtOH 96% (3 x 1 mL) and acetone (3 x 2 mL). Crude product was dissolved in absolute EtOH (40 mL) and filtered. The solvent was removed to yield pure 3. Yield: 11% (108 mg). ^1^H NMR (250 MHz, *d_6_*-DMSO): δ 10.86 (s, 1H), 10.26 (s, 1H), 7.89 (d, *J* = 2.2 Hz, 1H), 7.71 (dd, *J* = 8.6, 2.3 Hz, 1H), 6.93 (d, *J* = 8.6 Hz, 1H).

**3-formyl-4-hydroxybenzoic acid (7)** (Irfan et al., 2016). A solution of hexamethylenetetramine (7.5 g, 54.5 mmol, 1 eq) in TFA (20 mL) was added dropwise to 4-hydroxybenzoic acid (7.5 g, 54.0 mmol, 1 eq) in TFA (20 mL) under N_2_ atmosphere and refluxed overnight. The reaction mixture was then cooled to room temperature and HCl 4 M added (150 mL). The reaction mixture was stirred for 4 h at room temperature. The yellow precipitate was filtered off, washed with an excess of water and dissolved in MeOH. The MeOH solution was dried with anhydrous Na_2_SO_4_ and the solvent removed to obtain a white-yellowish solid. Yield: 44 % (3.99 g). ^1^H NMR (300 MHz, *d_6_*-DMSO): 12.75 (bs, 1H), 11.51 (bs, 1H), 10.30 (s, 1H), 8.25 (d, *J* = 2.3 Hz, 1H), 8.05 (dd, *J* = 8.7, 2.3 Hz, 1H), 7.09 (d, *J* = 8.7 Hz, 1H).

**3-formyl-4-(methoxymethoxy)benzoic acid (8).** A solution of chloromethyl methyl ether (3.7 mL, 49.7 mmol, 5.5 eq) in anhydrous DCM (120 mL) was added dropwise for 45 min to a solution of 7 (1.5 g, 9 mmol, 1 eq) and DIEA (8.6 mL, 49.7 mL, 5.5 eq) in anhydrous DCM (80 mL) at 0 °C. The resulting reaction mixture was stirred for 48 h at room temperature. Then, it was quenched with saturated NH_4_Cl (250 mL) and extracted with DCM (3x150 mL). The organic layer was dried under reduced pressure to provide the intermediate ether ester compound, a dark orange oil. To a solution of this crude ether ester in MeOH (500 mL), NaOH 15% (400 mL) was added at 0 C and then let it stir at room temperature during 3 h. Reaction progress was controlled by TLC. pH was then adjusted to 3 with HCl 6 M at 0 °C. The solution was extracted with EtOAc (2 x 250 mL). The organic layer was washed with water (2 x 50 mL) and brine (2 x 50 mL), and dried under reduced pressure to yield a white-yellowish solid. The solid was recrystallized in EtOAc to obtain pure 8. Yield: 59 % (1.1 mg). HR-MS (ESI^-^, MeOH) for [8-H]^-^ = 209.0450 (theoretical = 209.0444). ^1^H NMR (360 MHz, *d_6_*-DMSO): δ 13.05 (bs, 1H), 10.39 (s, 1H), 8.26 (d, *J* = 2.1 Hz, 1H), 8.17 (dd, *J* = 8.8, 2.1 Hz, 1H), 7.39 (d, *J* = 8.8 Hz, 1H), 5.45 (s, 2H), 3.46 (s, 3H). ^13^C NMR (400 MHz, *d_6_*-DMSO): δ 189.30, 166.68, 162.46, 137.13, 129.80, 125.01, 124.51, 115.99, 94.9, 56.82.

**(*E*)-3-((2-acetamidophenylimino)methyl)-4-(methoxymethoxy) benzoic acid (9).** A solution of compound 1 (108 mg, 0.71 mmol, 1.01 eq) in absolute EtOH (20 mL) was added dropwise to compound 8 (150 mg, 0.71 mmol, 1 eq) in absolute EtOH (20 mL) and molecular sieve. The reaction mixture was stirred for 24 h at room temperature. Then, it was filtered under vacuum and the filtrate dried under reduced pressure to render a yellowish solid. High purity was obtained after recrystallization with EtOAc. Yield: 96 % (236 mg). HR-MS (ESI^-^, MeOH) for [9-H]^-^ = 341.1138 (theoretical = 341.1132). ^1^H NMR (250 MHz, *d_6_*-DMSO): δ 12.93 (s, 1H), 9.21 (s, 1H), 8.86 (s, 1H), 8.70 (d, *J* = 2.0 Hz, 1H), 8.07 (dd, *J* = 8.7, 2.2 Hz, 1H), 8.01 (d, *J* = 7.6 Hz, 1H), 7.35 (d, *J* = 8.8 Hz, 1H), 7.28 – 7.10 (m, 3H), 5.43 (s, 2H), 3.45 (s, 3H), 2.09 (s, 3H). ^13^C NMR (400 MHz, *d_6_*-DMSO): δ 168.78, 167.21, 160.41, 155.76, 142.68, 134.42, 132.87, 129.82, 126.96, 125.09, 124.96, 124.80, 122.39, 118.69, 115.19, 94.83, 56.70, 24.41.

**(*S*)-2-amino-5-(3-(2,2,4,6,7-pentamethyl-2,3-dihydrobenzofuran-5-ylsulfonyl)guanidino)pentan-amide resin: Arg(Pbf)-Resin (10).** DCM (2 x 15 mL) was added to Rink Amide MBHA (2 g resin scale synthesis, 0.59 mmol/g, 1 eq) for 5 min. The solvent was filtered and the Fmoc group was removed with piperidine (20% in DMF, 2 x 20 mL) for 20 min each. Removal of the Fmoc group was monitored by TLC (hexane:Et_2_O, 1:1.5). The resin was filtered out to remove the excess of piperidine and washed with DMF (2 x 10 mL). A solution of Fmoc-Arg(Pbf)-OH (2.3 g, 3.54 mmol, 3 eq) and HBTU (1.3 g, 3.42 mmol, 2.9 eq) in DMF (6 mL) was then added to the resin in DMF (10 mL) and stirred for 1 min, after which DIEA (1.23 mL, 7.08 mmol, 6 eq) in NMP (2 mL) was also added. The mixture was stirred for 1.5 h at room temperature. The resin was filtered out and washed with DMF (3 x 5 mL) and DCM (3 x 5 mL). Fmoc protecting group was then removed with piperidine (20% in DMF, 2 cycles x 20 mL) for 20 min each cycle to obtain compound 10. To confirm the coupling, an aliquot of 10 was simultaneously cleaved and deprotected with a TFA/TIS/H_2_O (95/2.5/2.5, (v/v/v)) mixture for 2 h at room temperature. The resin was filtered out and rinsed with TFA. The filtrate and rinses were combined and concentrated with a nitrogen stream. After precipitation with cold Et_2_O, the crude was recovered by centrifugation, dissolved in water and lyophilized. Yield: > 90 %. Analytical HPLC (8-36% B in 20 min, 1 mL/min): R_t_ = 15 min. ^1^H NMR (300 MHz, D_2_O): δ 4.50 – 4.35 (m, 1H), 3.15 (t, *J* = 6.8 Hz, 2H), 2.13 (s, 1H), 1.99 – 1.73 (m, 2H), 1.72 – 1.55 (m, 2H).

**(*R*)-*N*-(1-amino-5-guanidino-1-oxopentan-2-yl)-3-formyl-4-hydroxybenzamide (4).** A solution of 9 (262 mg, 0.77 mmol, 1.3 eq) and HBTU (269 mg, 0.71 mmol, 1.2 eq) in DMF (6 mL) were added into 10 (1 g, 0.59 mmol/g, 1 eq) in DMF (8 mL). After 1 min of stirring, DIEA (198 mg, 1.53 mmol, 2.6 eq) in NMP (2 mL) was added and the mixture was stirred at room temperature for 1.5 h. The resin was filtered out and washed with DMF (3 x 5 mL) and DCM (3 x 5 mL), letting the resin dry. Then, it was simultaneously cleaved and deprotected with a TFA/TIS/H_2_O (95/2.5/2.5, (v/v/v)) mixture for 2 h at room temperature. The resin was filtered out and rinsed with TFA. The filtrate and rinses were combined and concentrated with a nitrogen stream. After precipitation from the filtrate with cold Et_2_O, the crude 4 was recovered by centrifugation, dissolved in water and lyophilized. It was purified through reversed-phase preparative HPLC in a C12 Axia column (250 mm x 21.20 mm, 4 µm, 90 Å) with the two-solvent gradient A and B to render pure product 4. Yield: 30 % (55 mg). Preparative HPLC (8-36% B in 30 min, 10 mL/min): R_t_ = 17.3 min. Analytical HPLC (8-36% B in 20 min, 1 mL/min): R_t_ = 11.4 min (purity ≥ 95%). HR-MS (ESI^+^, ACN-H_2_O) for [4+H]^+^ = 322.1520 (theoretical: 322.1510). ^1^H NMR (300 MHz, D_2_O): δ 9.99 (s, 1H), 8.18 (d, *J* = 2.3 Hz, 1H), 7.98 (dd, *J* = 8.8, 2.3 Hz, 1H), 7.08 (d, *J* = 8.8 Hz, 1H), 4.48 (m, 1H), 3.23 (t, *J* = 6.7 Hz, 2H), 2.11 – 1.61 (m, 4H). ^13^C NMR (400 MHz, *d_6_*-DMSO): δ 197.12, 176.68, 169.25, 162.75, 156.68, 135.79, 133.13, 125.00, 120.72, 117.65, 53.80, 40.42, 28.04, 24.49.

**R_9_(*)-Resin (18) and G_4_-R_9_(*)-Resin (11 and 12).** Fmoc-protected (in the *N*-terminus) 11 and 12 were automatically synthesized assisted by a Biotage Initiator+ Alstra synthesizer, and following the same procedure as for Arg(Pbf)-Resin (10). Fmoc deprotection was carried out by using piperidine (20% in DMF, 2 x 20 mL, 20 min) at room temperature to render 11 and 12. Peptide synthesis yield was considered > 90%. Aliquots of Fmoc-protected 11 and 12 were simultaneously cleaved and the side-chain protecting groups of the Arg residues removed with the TFA/TIS/H_2_O mixture. Following the same procedure as for the aliquot of 10, they were collected and lyophilized, and their purity assessed by analytical reversed-phase HPLC. For the NMR characterization, Fmoc deprotected peptide aliquots (11 and 12) were used, following the same protocol. From Fmoc-protected 11: Analytical HPLC (0-50%B in 30 min, 1 mL/min): R_t_ = 20.2 min (purity ≥ 95%). ^1^H NMR of Fmoc-deprotected (300 MHz, D_2_O): δ 4.42-4.21 (9H, bs), 3.29-3.10 (18H, bs), 1.9-1.5 (38H, m). From Fmoc-protected 12: Analytical HPLC (0-50%B in 30 min, 1 mL/min): R_t_ =18.6 min (crude purity > 90%). ^1^H NMR of Fmoc-deprotected (300 MHz, D_2_O): δ 4.31-4.10 (9H, bs), 3.95-3.7 (4H, m), 3.20-3.02 (18H, bs), 1.85-1.5 (38H, m).

**7 conjugated to R_9_ peptide (5).** A solution of 9 (66 mg, 0.192 mmol, 1.1 eq) and HBTU (70 mg, 0.184 mmol, 1.05 eq) in DMF (6 mL) were added into 11 (300 mg, 0.59 mmol/g, 1 eq) in DMF (8 mL). After 1 min of stirring, DIEA (52 mg, 0.403 mmol, 2.2 eq) in NMP (2 mL) was added and the mixture was stirred at room temperature for 1.5 h. The resin was filtered out and washed with DMF (3 x 5 mL) and DCM (3 x 5 mL). Then, it was simultaneously cleaved and deprotected with a TFA/TIS/H_2_O (95/2.5/2.5, (v/v/v)) mixture for 2 h at room temperature, following the same procedure as that for 4. The crude of 5 was purified through reversed-phase preparative HPLC. Yield: 15% (42 mg). Preparative HPLC (10-22% B in 20 min, 10 mL/min): R_t_ = 22.1 min. Analytical HPLC (0-50%B in 30 min, 1 mL/min): R_t_ = 15.0 min (purity ≥ 95%). ESI-MS (ESI^+^, H_2_O-MeOH) for [5+4H]^4+^ = 393.5 (theoretical: 393.5), [5+5H]^5+^ = 315.0 (theoretical: 315.0) and [5+6H]^6+^ = 262.7 (theoretical: 262.7). ^1^H NMR (360 MHz, D_2_O): δ 9.98 (s, 1H), 8.20 (s, 1H), 7.99 (d, *J* = 8.3 Hz, 1H), 7.10 (d, *J* = 8.2 Hz, 1H), 4.30 (s, 9H), 3.18 (s, 18H), 2.00 – 1.35 (m, 36H).

**7 conjugated to G_4_-R_9_ peptide (6).** A solution of 9 (330 mg, 0.964 mmol, 1.1 eq) and HBTU (352 mg, 0.929 mmol, 1.05 eq) in DMF (8 mL) were added into 12 (1.5 g, 0.59 mmol/g, 1 eq) in DMF (10 mL). After 1 min of stirring, DIEA (252 mg, 1.95 mmol, 2.2 eq) in NMP (2 mL) was added and the mixture was stirred at room temperature for 1.5 h. The resin was filtered out and washed with DMF (3 x 5 mL) and DCM (3 x 5 mL). Then, it was simultaneously cleaved and deprotected with a TFA/TIS/H_2_O (95/2.5/2.5, (v/v/v)) mixture for 2 h at room temperature, following the same procedure as that for 4. The crude of 6 was purified through reversed-phase preparative HPLC. Yield: 11% (37 mg). Preparative HPLC (10-23% B in 20 min, 10 mL/min): R_t_ = 19.6 min. Analytical HPLC (0-50% B in 30 min, 1 mL/min): R_t_ = 14.5 min (purity ≥ 95%). HR-MS (ESI^+^, H_2_O-MeOH) for [6+5H]^5+^ = 360.6182 (theoretical: 360.6150) and [6+3Na+2H+CH_3_OH]^5+^ = 380.2105 (theoretical: 380.2094). ^1^H NMR (400 MHz, D_2_O): δ 9.94 (s, 1H), 8.15 (d, *J* = 2.4 Hz, 1H), 7.94 (dd, *J* = 8.8, 2.4 Hz, 1H), 7.02 (d, *J* = 8.8 Hz, 1H), 4.26 – 4.12 (m, 9H), 4.08 (s, 2H), 3.97 – 3.74 (m, 6H), 3.14 – 2.99 (m, 18H), 1.80 – 1.42 (m, 36H).

**(*E*)-3-((2-acetamidophenylimino)methyl)-4-hydroxybenzene-sulfonic acid (H_2_L2).** Compound 3 (50 mg, 0.247 mmol, 1 eq) in absolute EtOH (20 mL) was dropwise added to a solution of 1 (45 mg, 0.297 mmol, 1.2 eq) in absolute EtOH (10 mL) at 0 ºC under stirring and with molecular sieve. The mixture was stirred for 15 min at 0 ºC and then overnight at room temperature. Molecular sieve was filtered off and the solvent of the filtrate removed to obtain crude H_2_L2. The product was washed with DCM (3 x 5 mL) to obtain pure ligand H_2_L2. Yield: 53% (43 mg). HR-MS (ESI^-^, MeOH) for [H_2_L2-H]^-^ = 333.0545 (theoretical = 333.0551). ^1^H NMR (360 MHz, d_6_-DMSO): δ 13.03 (s, 1H), 9.56 (s, 1H), 8.94 (s, 1H), 7.95 (s, 1H), 7.62 (d, J = 7.0 Hz, 2H), 7.46 – 7.36 (m, 1H), 7.31 – 7.19 (m, 2H), 6.90 (d, J = 8.3 Hz, 1H), 2.05 (s, 3H). ^13^C NMR (400 MHz, d_6_-DMSO): δ 168.94, 164.03, 161.24, 142.45, 140.48, 132.61, 131.21, 130.19, 127.53, 126.53, 125.88, 119.97, 118.92, 116.26, 24.38.

**(*R*,*E*)-3-((2-acetamidophenylimino)methyl)-*N*-(1-amino-5-guanidino-1-oxopentan-2-yl)-4-hydroxybenzamide (H_2_L3).** Compound 1 (13 mg, 0.087 mmol, 1.4 eq) in absolute EtOH (12 mL) was dropwise added to a solution of 4 (20 mg, 0.062 mmol, 1 eq) in absolute EtOH (10 mL) at 0 ºC under stirring and with molecular sieve. The mixture was stirred for 15 min at 0 ºC and then 24 h at room temperature. Molecular sieve was filtered off and the solvent of the filtrate removed to obtain crude H_2_L3. The crude was washed with EtOAc (3 x 5 mL) to obtain pure ligand H_2_L3. Yield: 68% (19 mg). HR-MS (ESI^+^, MeOH) for [H_2_L3+H]^+^ = 454.2198 (theoretical = 454.2197). ^1^H NMR (250 MHz, D_2_O): δ 9.97 (s, 1H), 8.08 (d, J = 2.4 Hz, 1H), 7.84 (dd, J = 8.9, 2.3 Hz, 1H), 7.18 – 7.07 (m, 1H), 7.02 (d, J = 6.5 Hz, 1H), 6.87 (d, J = 9.1 Hz, 1H), 6.80 (t, J = 7.7 Hz, 1H), 4.40 (m, 1H), 3.17 (t, J = 6.7 Hz, 2H), 2.12 (s, J = 5.6 Hz, 2H), 1.96 – 1.55 (m, 4H). ^13^C NMR (500 MHz, D_2_O): δ 192.82, 176.88, 173.94, 169.68, 162.79, 156.70, 142.01, 135.71, 132.04, 128.73, 127.63, 122.82, 121.65, 119.76, 117.76, 117.45, 115.15, 53.80, 40.45, 28.07, 24.55, 21.89.

**Ligand H_2_L1 conjugated to R_9_ peptide (H_2_L4).** Compound 1 (2 mg, 0.013 mmol, 2.1 eq) in absolute EtOH (5 mL) was dropwise added to a solution of 5 (10 mg, 0.0064 mmol, 1 eq) in absolute EtOH (5 mL) at 0 ºC under stirring and with molecular sieve. The mixture was stirred for 15 min at 0 ºC and then 24 h at room temperature. Molecular sieve was filtered off and the solvent of the filtrate removed. The crude was washed with EtOAc (3 x 5 mL), recovered with H_2_O and lyophilized to obtain pure ligand H_2_L4. Yield: 88% (9.6 mg). HR-MS (ESI^+^, MeOH) for [H_2_L4+4H]^4+^ = 426.5133 (theoretical = 426.5126). ^1^H NMR (400 MHz, D_2_O): δ 10.08 (s, 1H), 8.02 (s, 1H), 7.73 (d, J = 8.3 Hz, 1H), 7.14 (t, J = 7.5 Hz, 1H), 7.04 (d, J = 7.0 Hz, 1H), 6.89 (d, J = 6.7 Hz, 1H), 6.82 (t, J = 6.5 Hz, 1H), 6.67 (d, J = 7.8 Hz, 1H), 4.26 (s, 9H), 3.13 (s, 18H), 2.14 (s, 3H), 1.85 – 1.50 (m, 36H).

**Ligand H_2_L1 conjugated to G_4_-R_9_ peptide (H_2_L5)**. Compound 1 (2 mg, 0.013 mmol, 2.4 eq) in absolute EtOH (5 mL) was dropwise added to a solution of 6 (20 mg, 0.053 mmol, 1 eq) in absolute EtOH (5 mL) at 0 ºC under stirring and with molecular sieve. Pure ligand H_2_L5 was obtained following the same protocol as for H_2_L4. Yield: 8.0 mg (78%). HR-MS (ESI^+^, MeOH) for [H_2_L5+4H]^4+^ = 483.5343 (theoretical = 483.5341), for [L7+5H]^5+^ = 387.0305 (theoretical = 387.0287). ^1^H NMR (500 MHz, D_2_O): δ 10.08 (s, 1H), 8.08 (s, 1H), 7.78 (d, J = 9.0 Hz, 1H), 7.20 (t, J = 7.6 Hz, 1H), 7.10 (d, J = 7.8 Hz, 1H), 6.95 (d, J = 8.0 Hz, 1H), 6.88 (t, J = 7.5 Hz, 1H), 6.71 (d, J = 9.0 Hz, 1H), 4.37 – 4.24 (m, 9H), 4.17 – 3.84 (m, 8H), 3.28 – 3.06 (m, 18H), 2.20 (s, 3H), 1.88 – 1.52 (m, 36H).

**Complex C2.** A solution of H_2_L2 (22 mg, 0.066 mmol, 1 eq) and NaOAc (5.4 mg, 0.066 mmol, 1 eq) in MeOH (4 mL) was dropwise added to a solution of Cu(OAc)_2_·H_2_O (13.15 mg, 0.066 mmol, 1 eq) in MeOH (4 mL) at 0 ºC under stirring. The mixture was stirred for 24 h at room temperature. Yield: 73% (19 mg). The precipitate was filtered off and washed with MeOH (3 x 1 mL) and DCM (3 x 3 mL). HR-MS (ESI^+^, MeOH-DMSO) for [Cu(L2)+Na]^+^ = 417.9646 (theoretical = 417.9655).

**Complex C3.** A solution of Cu(OAc)_2_·H_2_O (6.6 mg, 0.033 mmol, 1.5 eq) in ACN (5 mL) was added to a solution of H_2_L3 (10 mg, 0.022 mmol, 1 eq) in MeOH (5 mL) under stirring. The mixture was stirred for 24 h at room temperature. Solvent was removed under reduced pressure and the solid washed with ACN (4 x 5 mL). Yield: 78% (8 mg) HR-MS (ESI^+^, MeOH) for [Cu(L3)+H]^+^ = 515.1329 (theoretical = 515.1337).

**Complex C4.** A solution of Cu(OAc)_2_·H_2_O (1.1 mg, 0.0055 mmol, 1.9 eq) in ACN (5 mL) was added to a solution of H_2_L4 (5 mg, 0.0029 mmol, 1 eq) in MeOH (5 mL) under stirring. The mixture was stirred for 24 h at room temperature. Solvent was removed under reduced pressure and the solid washed with ACN (4 x 5 mL). Yield: 88% (4.6 mg). HR-MS (ESI^+^, H_2_O-MeOH) for [Cu(L4)+2H]^2+^ = 882.4696 (theoretical = 882.4749), for [Cu(L4)+MeOH+3Na]^3+^ = 621.3068 (theoretical = 621.3097), for [Cu(L4)+3H]^3+^ = 588.6500 (theoretical =588.6524), for [Cu(L4)+4H]^4+^ = 441.7483 (theoretical = 441.7411).

**Complex C5.** A solution of Cu(OAc)_2_·H_2_O (1.1 mg, 0.0055 mmol, 2 eq) in ACN (2.5 mL) was added to a solution of H_2_L5 (5 mg, 0.0026 mmol, 1 eq) in MeOH (2.5 mL) under stirring. The mixture was stirred for 24 h at room temperature. Solvent was removed under reduced pressure and the solid washed with ACN (4 x 5 mL). Yield: 90% (4.8 mg) HR-MS (ESI^+^, H_2_O-MeOH) for [Cu(L5)+3H]^3+^ = 664.6829 (theoretical = 664.6810), for [Cu(L5)+CH_3_OH+H+3Na]^4+^ = 523.2568 (theoretical = 523.2566), for [Cu(L5)+4H]^4+^ = 498.7642 (theoretical = 498.7626), for [Cu(L5)+CH_3_OH+2H+3Na]^5+^ = 418.8074 (theoretical = 418.8059), for [Cu(L5)+5H]^5+^ = 399.2147 (theoretical = 399.2115).

1. **Characterization data (NMR, UV-vis, MS)**

**Figure S1.** ^1^H NMR (360 MHz, *d_6_*-DMSO, top) and ^13^C NMR (400 MHz, *d_6_*-DMSO, down) spectra of compound **8**.

**Figure S2.** ^1^H NMR (250 MHz, *d_6_*-DMSO, top) and ^13^C NMR (400 MHz, *d_6_*-DMSO, down) spectra of compound **9**.

**Figure S3.** ^1^H NMR (300 MHz, D_2_O) spectrum of a cleaved and deprotected aliquot of compound **10**.

**Figure S4.** ^1^H NMR (300 MHz, *d_6_*-DMSO, top) and ^13^C NMR (400 MHz, *d_6_*-DMSO, down) spectra of compound **4**.

**Figure S5.** ^1^H NMR (300 MHz, D_2_O) spectra of a cleaved and deprotected aliquots of compound **11** (top) and **12** (down).

**Figure S6.** ^1^H NMR (360 MHz, D_2_O, top) spectrum of compound **5** and ^1^H NMR (400 MHz, D_2_O, down) spectrum of compound **6.** The remaining H_2_O signal was suppressed for clarity.

**Figure S7.** ^1^H NMR (360 MHz, *d_6_*-DMSO, top) and ^13^C NMR (400 MHz, *d_6_*-DMSO, down) spectra of ligand **H_2_L2**.

**Figure S8.** ^1^H NMR (250 MHz, D_2_O, top) and ^13^C NMR (500 MHz, D_2_O, down) spectra of ligand **H_2_L3**.

**Figure S9.** ^1^H NMR (400 MHz, D_2_O, top) spectrum of **H_2_L4** (top) and ^1^H NMR (500 MHz, D_2_O, down) spectrum of **H_2_L5** (down).

**Figure S10.** UV-vis spectra of complexes **C2** and **C3** in DMSO at 1 mM (**A** and **B**, respectively; Cu(II) *d-d* bands), as compared to the corresponding ligands (**H_2_L2** and **H_2_L3**, respectively) and Cu(II) acetate control; and **C2** and **C3** at 50 µM (MLCT bands, **C**), as compared to the parent complex **C1**.

| **A** | ***HR-MS*** | | |
| --- | --- | --- | --- |
| **C2** |  | **C4** |  |
| **C3** |  | **C5** |  |

**Figure S11.** HR-MS (ESI^+^, MeOH or MeOH-H_2_O) for the corresponding Cu(II) complexes **C2-C5**, confirming the successful complexation.

**
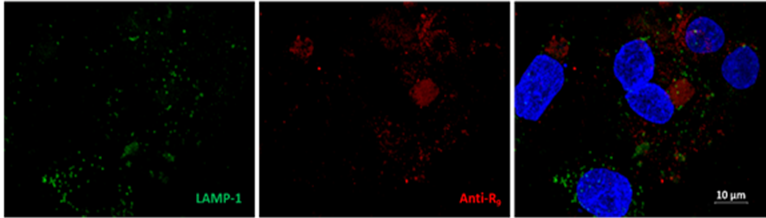
**

**Figure S12.** Cellular distribution of complex **C4** after 4h incubation in HeLa cancer cells. HeLa cells were fixed and stained with anti-LAMP-1 (green, for lysosomes) and Anti-R_9_ (red) antibodies, and DAPI (blue, nuclei). Last column shows the merged images.

1. **References**

Irfan, R. M., Jiang, D., Sun, Z., Lu, D., & Du, P. (2016). Enhanced photocatalytic H2 production on CdS nanorods with simple molecular bidentate cobalt complexes as cocatalysts under visible light. *Dalton Transactions*, *45*(32), 12897–12905. https://doi.org/10.1039/c6dt02148d

Kirker, G. W. (1980). An efficient synthesis of 5-sulfosalicylaldehyde. *Organic Preparations and Procedures International*, *12*(3–4), 246–249. https://doi.org/10.1080/00304948009458562

Shirin, Z., Thompson, J., Liable-sands, L., Yap, G. P. A., Rheingold, L., & Borovik, A. S. (2002).*2 -Symmetric ligands containing hydrogen bond donors: synthesis and properties of Cu(II) complexes of 2,6-bis[*N,N´-*(2-carboxamidophenyl)carbamoyl]pyridine. *J. Chem. Soc., Dalton Trans.*, 1714–1720. https://doi.org/10.1039/b107166c
